# Predicting presence and absence of trout (*Salmo trutta*) in Iran

**DOI:** 10.1016/j.limno.2013.12.001

**Published:** 2014-03

**Authors:** Hossein Mostafavi, Florian Pletterbauer, Brian W. Coad, Abdolrassoul Salman Mahini, Rafaela Schinegger, Günther Unfer, Clemens Trautwein, Stefan Schmutz

**Affiliations:** a Department of Water, Atmosphere and Environment, Institute of Hydrobiology and Aquatic Ecosystem Management, BOKU University of Natural Resources and Life Sciences, Vienna, Austria; b Department of Bio-diversity and Ecosystem Management, Environmental Sciences Research Institute, Shahid Beheshti University, Tehran, Iran; c Canadian Museum of Nature, Ottawa, Canada; d Faculty of Fishery and Environment, Gorgan University of Agricultural Sciences and Natural Resources, Gorgan, Iran

**Keywords:** Brown trout, Species distribution modelling, Iran

## Abstract

Species distribution modelling, as a central issue in freshwater ecology, is an important tool for conservation and management of aquatic ecosystems. The brown trout (*Salmo trutta*) is a sensitive species which reacts to habitat changes induced by human impacts. Therefore, the identification of suitable habitats is essential. This study explores the potential distribution of brown trout by a species distribution modelling approach for Iran. Furthermore, modelling results are compared to the distribution described in the literature. Areas outside the currently known distribution which may offer potential habitats for brown trout are identified. The species distribution modelling was based on five different modelling techniques: Generalised Linear Model, Generalised Additive Model, Generalised Boosting Model, Classification Tree Analysis and Random Forests, which are finally summarised in an ensemble forecasting approach. We considered four environmental descriptors at the local scale (slope, bankfull width, wetted width, and elevation) and three climatic parameters (mean air temperature, range of air temperature and annual precipitation) which were extracted on three different spatial extents (1/5/10 km). The performance of all models was excellent (≥0.8) according to the TSS (True Skill Statistic) criterion. Slope, mean and range of air temperature were the most important variables in predicting brown trout occurrence. Presented results deepen the knowledge about distribution patterns of brown trout in Iran. Moreover, this study gives a basic background for the future development of assessment methods for riverine ecosystems in Iran.

## Introduction

Iran is the second largest country in Southwest Asia (1,648,195 km^2^), and is larger than France, Germany and Spain together. The country lies in the Palearctic zoogeographical realm bordering the Oriental and African ones (Coad and Vilenkin, 2004), and features a great diversity of aquatic species. Overall, the ichthyofauna of Iran comprises a total of 203 species (180 native of which 40 are endemic and 23 exotic species) (Esmaeili et al., 2010, Teimori et al., 2012). Freshwaters are already exposed to numerous anthropogenic stressors, and are naturally fragmented in stream networks or intermittent water bodies. One of the major human impacts on Iranian rivers is the poor water quality due to urbanization, agriculture and industrial activities (Coad, 1980, Kiabi et al., 1999a, Kiabi et al., 1999b, Esmaeili et al., 2007). Other impacts are associated with changes in hydrology, restricted water recourses, increasing hydropower plant constructions and introduced species (Mostafavi, 2007, Abdoli and Naderi, 2009). All impacts collectively resulted in seven fish species categorized as endangered (EN), and five as vulnerable (VU). Most likely, many other fish species have not been included in this classification due to insufficient data (Esmaeili et al., 2007). Therefore, modelling freshwater fish distributions seems particularly important to implement management and conservation strategies (Dauwalter and Rahel, 2008, Logez and Pont, 2011) especially for sensitive species like brown trout which have already declined in their original distribution.

This study aims to develop a framework for accurate predictive distribution models for brown trout (*Salmo trutta*) as a model species for further biological assessment activities in Iranian rivers which, to our knowledge, has not been done so far. Brown trout as an indicator species shows sensitivity to a variety of human pressures (e.g. water pollution, habitat degradation). Normally, it inhabits headwaters with high oxygen saturation, steep slope, fast flow, suitable temperature regimes and adequate food (Elliott, 1994, Abdoli, 2000). Due to anthropogenic influences, the brown trout was eliminated from many original habitats in Iran (Coad, 2013). Although a basic evaluation of the species’ distribution based on expert judgment exists, a quantitative evaluation based on a statistical approach is missing. Therefore, this study aims at building a species distribution model (SDM) to find the potential distribution of brown trout for Iran.

Brown trout shows a wide distribution and is recorded from all over Europe, northern Africa, and western Asia (i.e. from the British Isles to western Siberia, and from the Atlas Mountains in North Africa to the glacial streams of Iceland) (MacCrimmon and Marshall, 1968). Current occurrences of brown trout in Iran are reported from the Caspian Basin in the north, from the Urmia Basin in the north-west, and the endorheic Namak Basin in the north-central region of Iran (Abdoli, 2000, Abdoli and Naderi, 2009, Coad, 2013). However, based on the different literature sources describing the historical zoogeography of the basins (Bernatchez, 2001), the distribution of brown trout (Heckel, 1843, Walczak, 1972), and the phylogenetic relationships between different populations (e.g. Bernatchez, 2001, Hashemzadeh et al., 2012), it might be reasonable that brown trout also occurs in other Iranian basins (e.g. Tigris Basin). Even if the species is absent, it is unclear whether natural physical barriers (e.g. geologic history), anthropogenic activities, or climatic changes triggered its absence in those regions. Furthermore, with the exception of its current known distribution, little information is available concerning the potential of other basins to be inhabited by brown trout populations. Therefore, this study investigates the potential distribution over the whole extent of Iran.

Species distribution modelling has been a central issue in ecology in recent years (Guisan and Thuiller, 2005). An increasing number of studies in ecology, biogeography, and conservation biology have tried to build predictive models of species distribution, aiming at a better protection and management of natural resources and ecosystems. In stream fish ecology, there have been studies assessing impacts of habitat alteration (Logez and Pont, 2011), estimates on habitat suitability for species re-introductions (Lek et al., 1996), predicting the likelihood of species invasions (Poulos et al., 2012), examining the influence of scale and geography or relationships between fishes and landscape variables (Pont et al., 2005), identifying areas of persistence for threatened or endangered species (Dauwalter and Rahel, 2008) and finally, demonstrating the utility of species distribution modelling to guide conservation management of stream fishes (Filipe et al., 2013).

Various statistical methods are used to model species distributions in the field of freshwater ecology (e.g. Lek and Guégan, 1999, Pont et al., 2005, Buisson et al., 2008). All modelling techniques relate the observed distribution of a species to several environmental variables (Austin, 2007, Elith and Leathwick, 2009). Nevertheless, some authors (e.g. Elith and Graham, 2009, Thuiller et al., 2009b) have demonstrated large discrepancies between different techniques, thus making the choice of an appropriate approach even more difficult. The results of different models are not only dependent on the relationship between species occurrence and environmental conditions (linear or nonlinear) but also on the used dataset, i.e. information on presence and absence (Elith and Graham, 2009). Accordingly, summarising different model types into an ensemble forecasting approach reduces uncertainty of individual techniques (Araújo and New, 2007). Both local and regional environmental variables can be useful for predicting species presence/absence. However, selection of environmental variables primarily depends on the ecological and biophysical processes influencing the biota. Practically, the availability of data as well as the purpose and requirements of the applied models (Austin, 2007) guide the variable selection. Hence, we test the suitability of available parameters characterising local and regional conditions to evaluate their ability to predict the distribution of brown trout in Iran.

The objectives of this study are: (1) development of a robust statistical framework to predict brown trout distribution in Iran, (2) comparison of model performances over the extent of Iran, and (3) characterisation of the environmental predictors and their importance in the models on the Iranian extent.

## Materials and methods

### Study area

**Fig. 1:**
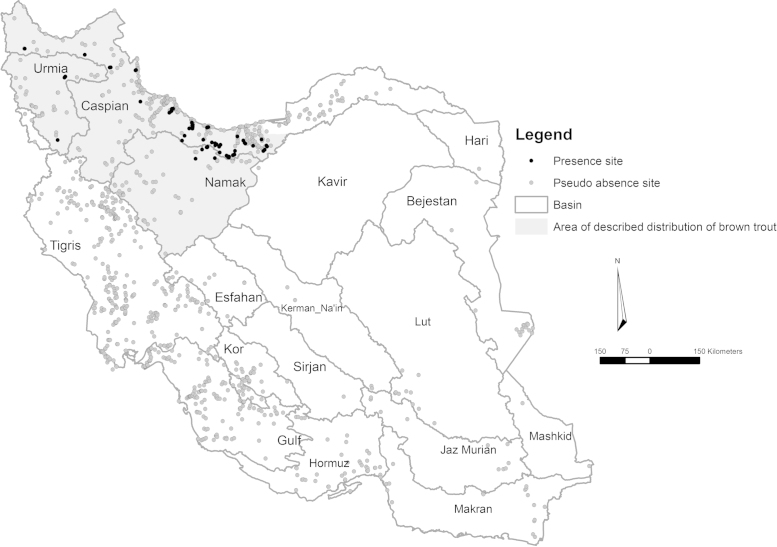
Distribution of study sites with occurrence data used in the modelling of brown trout in different freshwater basins plus the distribution of brown trout in Iran as described in the literature.

The study area was the country of Iran which encompasses 19 river basins (Coad, 1980) (Fig. 1). Iran's climate is classified as arid to semi-arid and more than 80% of the country has less than 250 mm annual rainfall. Mountain ranges block off the interior of Iran, where conditions are extremely continental. The narrow littoral zones on the Caspian shore and the Persian Gulf are more humid. Rain falls mainly from November to May, although the level is much higher in the Caspian littoral zone and much less in the interior plateau (Coad, 2013).

### Fish data

Occurrence data for brown trout covering several time periods were collected from two main data sources: (1) collated databases originating from previous field samplings, from several museums as well as from the literature containing actual and historical information (e.g. Berg, 1949, Saadati, 1977) and (2) our own field sampling data recorded in 2011 for validation. The primary database contained around 1700 sites which were reduced to 1090 sites after a detailed quality check concerning the reliability of the biological as well as the spatial information. All sites with an unclear position to the river network, outside the temporal period between 1950 and 2000, stocked with brown trout population and located in lakes and wetlands were excluded. In the dataset, positive occurrences of brown trout were limited to the Caspian, Urmia and Namak basins (Fig. 1).

We sampled 15 randomly and accessible trout absence sites plus 15 randomly and accessible sites with confirmed trout occurrence for the validation in early autumn of 2011, using single pass electric fishing (e.g. CEN, 2003). Length of the sampling site was calculated as 10–20 times the river width and overall at least a distance of 100 m was sampled to cover all available habitat types (i.e. riffles, runs, pools) (e.g. EFI+ Consortium, 2009). We established one stop net in the upstream reach and sampled the whole stream width with one (≤5 m wetted width) or two anodes (>5 m wetted width) followed by one or two hand-netters. The sampling effort moved slowly upstream to cover the habitat with a sweeping movement of the anodes, while attempting to draw fish out of hiding (EFI+ Consortium, 2009). The stunned fish were collected by two persons who accompanied the electric fishing team. Finally, after the identification the fish were released back into the stream.

### Natural environmental variables

We calculated the following variables to describe environmental conditions at the sampling sites: elevation (ELE), stream slope (SLO), wetted width (W_WID), bankfull width (B_WID), maximum air temperature (Max_TEM), minimum air temperature (Min_TEM), mean air temperature (A_TEM), the range of air temperature (R_TEM) and annual precipitation (PRE). As a catchment layer similar to CCM2 (Catchment Characterization and Modelling database; Vogt et al., 2003, Vogt et al., 2007, de Jager and Vogt, 2010) is not available for Iran, we therefore extracted ELE, W_WID and B_WID from Google Earth (Google Inc. 2009, Version 5), as Iran has different climates the water level of rivers is considerably affected and therefore two types of width could be recognisable. B_WID was the potential maximum width of the main river channel, typically marked by a change in vegetation, topography, or texture of sediment. SLO was calculated in a 1 km stretch for each site. Climate variables were extracted from WorldClim data (Hijmans et al., 2005, Hijmans et al., 2007) to characterise annual climate trends based on records for 50 years of monthly means (1950–2000), and interpolated at 30 arc-seconds grid extent (approximately 1 km at the Equator). Climate variables were extracted in circular buffers around each sampling site in three different size classes (1, 5 and 10 km) which hereafter are called small, medium and large extent respectively in the text. Climate processes can act on multiple scales, and we used these different buffer sizes to test whether effects were strongest at the small, medium and large extent. The other variables were calculated only at the site scale. Variable redundancy within environmental variables was tested by Spearman's rank correlation (*r*). If two variables were highly correlated (*r* > |0.75|) (Filipe et al., 2013), one of them was excluded to avoid co-linearity.

### Modelling techniques and ensemble forecasting

In this study the BIOMOD (BIOdiversity MODelling) package (Thuiller, 2003) was used within the R software (R Development Core Team, 2011). These tools enabled the examination of methodological uncertainties and the maximization of predictive performance of the SDMs (Thuiller et al., 2009a). This study compared the following five modelling techniques: (1) Generalised linear model (GLM) (McCullagh and Nelder, 1989), performed with polynomial terms (Pont et al., 2005, Logez et al., 2012) using a stepwise procedure to select the most significant variables based on the Akaike information criterion (AIC) (Akaike, 1974). (2) Generalised additive model (GAM) (Hastie and Tibshirani, 1990), performed with automatically selected smooth splines as a nonparametric extension of GLM to capitalise on the strengths of GLM without requiring the problematic steps of postulating a specific parametric response function. As for GLM, a stepwise procedure using the AIC was used to select the most parsimonious model. (3) Classification tree analysis (CTA) (Breiman et al., 1984), used with an internal 10-fold cross-validation to select the best trade-off between the number of leaves of the tree and explained deviance (Thuiller, 2003). CTA provides a good alternative to regression techniques, because it does not rely on an *a priori* hypothesis on the relationship between independent and dependent variables. (4) Generalised boosting models (GBM) (or boosting regression trees, BRT) (Friedman et al., 2000, Friedman, 2001), performed with a maximum number of 3000 trees and internal 10-fold cross-validation (Marmion et al., 2009). GBM are highly efficient at fitting data that are non-parametric (Ridgeway, 1999). (5) Random forests (RF) (Breiman, 2001) are a combination of tree predictors such that each tree depends on the values of a random vector sampled independently and with the same distribution for all trees in the forest. Random forests are actually a learning ensemble consisting of a bagging of un-pruned decision tree learners with a randomized selection of features at each split. Finally, all five modelling techniques were combined in an ensemble-forecasting framework as recommended by Araújo and New (2007).

### Pseudo-absence method

This study is based on a heterogeneous data set containing information from several sources (see Section “Fish data”). Due to varying sampling methods and investigation targets of compiled original datasets, the absence of brown trout could not be verified in all sites where the species was not recorded. Accordingly, sites that had no records for brown trout were not directly considered as actual absence in the models but build the basis for a repeated pseudo-absence selection in the modelling procedure. False absences can decrease the reliability of prediction models (Chefaoui and Lobo, 2008), and consequently, we used the “pseudo-absence”-approach. The pseudo-absence dataset is created during the model calibration by a random selection of a given number of points with a potential absence, i.e. points where the species was not recorded. This random selection was repeated ten times to cover different gradients in the dataset of pseudo-absences (Thuiller et al., 2009a, Barbet-Massin et al., 2012).

### Model calibration and evaluation

In contrast to the prevalence of the whole dataset (0.057), the prevalence in the model calibration was 0.33 (67 presences and 200 pseudo-absences) to ensure the stability of the modelling framework (Barbet-Massin et al., 2012). Firstly, model evaluation was based on different criteria: (1) the True Skill Statistic (TSS) which corresponds to the sum of sensitivity and specificity minus 1, and is independent to prevalence (Lobo et al., 2008), (2) the sensitivity (‘true positives’) and (3) specificity (‘true negatives’) (Thuiller et al., 2009a, Barbet-Massin et al., 2012). Additionally, we applied a cross-validation procedure by randomly splitting the data into calibration (80% of the data) and validation (20%) data sets with 10 repetition runs to assess the stability of the model performance.

Finally, all five modelling techniques were combined in an ensemble-forecasting framework as recommended by Araújo and New (2007). The ensemble was built out of all modelling techniques with a weighting factor (decay = 1.6), giving higher importance to models with a better performance according to the TSS criterion (Thuiller et al., 2009a). Variable importance was calculated by a permutation procedure used in BIOMOD, which is independent of the modelling technique (Thuiller et al., 2009a). Once the models are trained (i.e. calibrated), a standard prediction is made. Then, one of the variables is randomized and a new prediction is made. The correlation score between the new prediction and the standard prediction is calculated and gives an estimation of the variable importance in the model (Thuiller et al., 2009a).

**Fig. 2:**
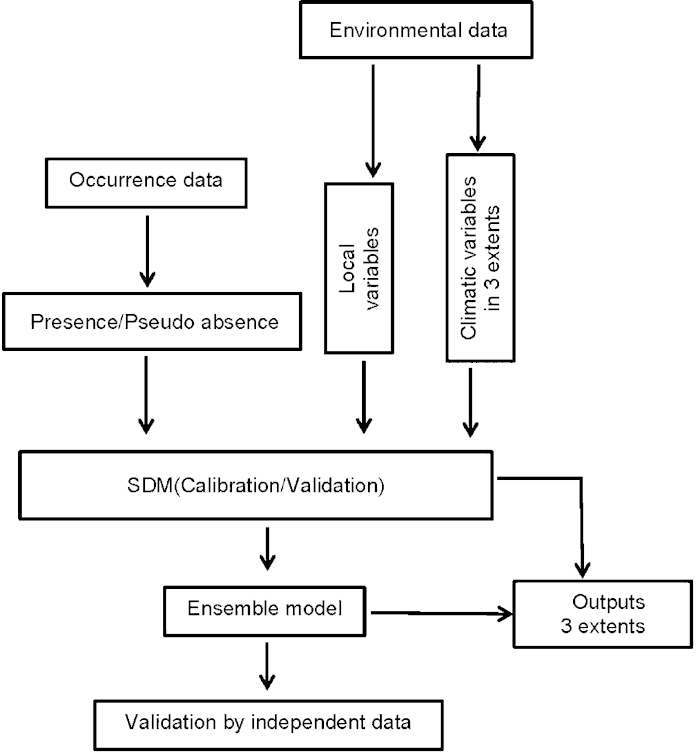
Workflow for the modelling framework to predict brown trout distribution in Iran.

We used the software ArcGIS Desktop 9.3 (ESRI© 1999–2008) to map the final results and to show the spatial pattern of brown trout distribution in Iran as presence and absence. According to the predicted brown trout occurrences, we then calculated the suitable range of the environmental variables (mean, max and min). Finally, the models were validated with an independent data set from own field sampling (see Section “Fish data”) containing 15 trout absence sites plus 15 sites with confirmed trout occurrence. Fig. 2 shows the workflow of the modelling framework to predict brown trout occurrence.

## Results

Brown trout was recorded at 63 sites out of the 1090 sites (Fig. 1). After correlation analyses (Table 1) seven environmental parameters (B_WID, W_WID, SLO, ELE, A_TEM, R_TEM, and PRE) remained as independent variables for the modelling. Their characteristics are described in Table 2.

**Table 1:** Matrix of Spearman rank correlations of environmental variables (*N* = 1090). The upper numbers are Spearman correlation coefficients and the lower numbers are *P* values. Correlations of *r* > |0.75| are shown in bold.

		W_WID	SLO	ELE	Max_TEM	Min_TEM	A_TEM	R_TEM	PRE
Small extent	B_WID	0.74	−0.26	−0.25	0.44	0.43	0.44	−0.01	−0.21
		0.00	0.00	0.00	0.00	0.00	0.00	0.77	0.00
Medium extent					0.43	0.43	0.43	−0.01	−0.20
					0.00	0.00	0.00	0.66	0.00
Large extent					0.44	0.44	0.44	−0.01	−0.21
					0.00	0.00	0.00	0.65	0.00

Small extent	W_WID		−0.34	−0.45	0.39	0.45	0.43	−0.13	0.00
			0.00	0.00	0.00	0.00	0.00	0.00	0.97
Medium extent					0.39	0.45	0.44	−0.14	0.01
					0.00	0.00	0.00	0.00	0.75
Large extent					0.39	0.46	0.44	−0.14	0.00
					0.00	0.00	0.00	0.00	0.89

Small extent	SLO			0.47	−0.38	−0.41	−0.42	0.05	0.08
				0.00	0.00	0.00	0.00	0.14	0.01
Medium extent					−0.41	−0.44	−0.46	0.06	0.06
					0.00	0.00	0.00	0.04	0.05
Large extent					−0.38	−0.41	−0.42	0.05	0.07
					0.00	0.00	0.00	0.13	0.01

Small extent	ELE				−0.41	−0.74	−0.62	0.64	−0.25
					0.00	0.00	0.00	0.00	0.00
Medium extent					−0.44	−0.74	−0.66	0.65	−0.25
					0.00	0.00	0.00	0.00	0.00
Large extent					−0.40	−0.73	−0.61	0.64	−0.25
					0.00	0.00	0.00	0.00	0.00

Small extent	Max_TEM					**0.83**	**0.94**	0.20	−0.49
						0.00	0.00	0.00	0.00
Medium extent						**0.85**	**0.94**	0.16	−0.46
						0.00	0.00	0.00	0.00
Large extent						**0.83**	**0.94**	0.21	−0.49
						0.00	0.00	0.00	0.00

Small extent	Min_TEM						**0.95**	−0.33	−0.28
							0.00	0.00	0.00
Medium extent							**0.97**	−0.34	−0.27
							0.00	0.00	0.00
Large extent							**0.95**	−0.32	−0.28
							0.00	0.00	0.00

Small extent	A_TEM							−0.09	−0.39
								0.00	0.00
Medium extent								−0.14	−0.35
								0.00	0.00
Large extent								−0.09	−0.39
								0.00	0.00

Small extent	R_TEM								−0.36
									0.00
Medium extent									−0.34
									0.00
Large extent									−0.36
									0.00

*Abbreviations*: B_WID, bankfull width; W_WID, wetted width; SLO, stream slope; A_TEM, mean air temperature; PRE, annual precipitation; ELE, elevation; R_TEM, range of air temperature.

**Table 2:** Mean and range (minimum–maximum) of environmental variables at different extents.

		B_ WID (m)	W_WID (m)	SLO (‰)	ELE (m)
	Mean	92.9	32.5	1.7	762
	Range	1.0–3539.8	1.0–608.6	0.0–28.0	(−)27–2708

		A_TEM ( °C)	R_TEM ( °C)	PRE (mm)	
Small extent	Mean	17.2	12.7	439	
	Range	5.5–27.5	6.9–16.5	53–1488	

Medium extent	Mean	17.6	12.8	441	
	Range	5.5–27.5	6.9–16.5	53–1489	

Large extent	Mean	17.7	12.9	442	
	Range	5.5–27.5	6.9–16.6	53–1490	

*Abbreviations*: B_WID, bankfull width; W_WID, wetted width; SLO, stream slope; ELE, elevation; A_TEM, mean air temperature; R_TEM, range of air temperature; PRE, annual precipitation.

Changing the extent of climate variables had no strong influence on the model performance (Table 3). The TSS, the sensitivity and the specificity of each single model among all extents as well as the average of the models within each extent were ‘excellent’ (i.e. ≥0.8 for TSS and >82% for sensitivity and specificity) (Table 3). The GLM had an inferior performance compared to the four other techniques (i.e. <0.81 in TSS), whereas RF had the highest performance values in all extents (i.e. >0.97 in TSS) (Table 3).

**Table 3:** Prediction accuracy measured using sensitivity, specificity, and TSS for all extents in pseudo-absence method.

	Model	Sensitivity (%)	Specificity (%)	TSS
Small extent	CTA	96.7	91.0	0.88
	GAM	95.4	87.8	0.83
	GBM	95.2	90.0	0.85
	GLM	96.5	83.3	0.80
	RF	99.0	99.0	0.98
	Average	96.6	90.2	0.87

Medium extent	CTA	95.1	91.3	0.86
	GAM	97.5	85.3	0.83
	GBM	95.9	88.6	0.85
	GLM	97.9	82.4	0.80
	RF	99.0	98.9	0.98
	Average	97.1	89.3	0.86

Large extent	CTA	96.9	88.9	0.86
	GAM	96.3	87.4	0.84
	GBM	96.6	87.9	0.85
	GLM	95.9	84.6	0.80
	RF	99.0	98.5	0.98
	Average	96.8	89.2	0.86

**Fig. 3:**
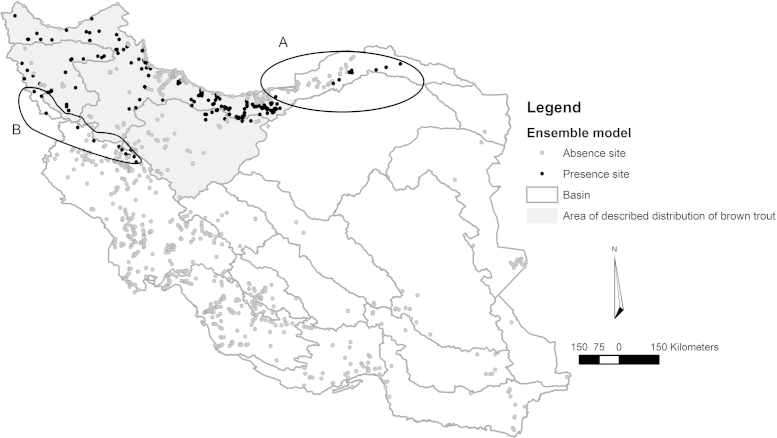
Predicted distribution of brown trout according to the ensemble model based on “large extent” climate variables: (A) predicted sites outside of the described distribution area in the eastern part of Caspian Basin and (B) predicted sites outside of the described distribution area in Tigris Basin.

In total, most occurrences were predicted for the Caspian, Urmia and Namak basins. The spatial pattern of predicted brown trout presences was coherent with the described distribution area (based on the literature) and showed similar results for the different extents of the climatic variables. As a representative example, Fig. 3 shows the predictions from the ensemble model using the large extent of climate variables. However, the models also identified potential sites outside the known distribution area. Those sites were found in the Tigris Basin and in the eastern part of the Caspian Basin (Fig. 3).

The relative importance of the environmental predictors did not show significant differences between different extents in average (Table 4). The variables SLO, A_TEM and R_TEM had the highest importance values (>18%), whereas B_WID, W_WID, ELE and PRE showed the lowest values (<6%). Nonetheless, the relative importance of variables was different among the different models in each extent (see Table 4, data only for large extent are shown because it was the same for other extents). As the results of large extent show in Table 4, in GBM and RF models the variables SLO, A_TEM and R_TEM had highest values (≥10%), whereas SLO, A_TEM, R_TEM and PRE were more important (>10%) in GAM and CTA models. Finally, A_TEM and R_TEM were most important (≥30%) for GLM. Therefore, each model was dominated by two to four environmental predictors and each extent in average was dominated by three variables. The variables B_WID, W_WID and ELE were of low importance (<6%) in all models (Table 4).

**Table 4:** Relative importance (in percentage) of environmental variables for each extent and all models.

	Model type	B_WID	W_WID	SLO	ELE	A_TEM	R_TEM	PRE
Small extent	Ensemble	0.9	0.2	42.4	2.4	28.8	24.6	0.7
Medium extent	Ensemble	2.3	4.9	27.3	1.1	35.2	27.2	2.0
Large extent	Ensemble	1.9	0.3	27.4	0.6	44.6	19.7	5.6

Large extent	CTA	5.9	0.0	35.4	0.0	23.0	22.3	13.5
	GAM	0.0	0.0	12.8	0.0	53.0	23.2	11.0
	GBM	0.4	0.4	40.4	0.4	45.8	12.0	0.5
	GLM	0.0	0.0	2.3	0.0	67.6	30.1	0.0
	RF	3.2	1.1	45.9	2.4	33.4	10.8	3.2

*Abbreviations*: B_WID, bankfull width; W_WID, wetted width; SLO, stream slope; A_TEM, mean air temperature; PRE, annual precipitation; ELE, elevation; R_TEM, range of air temperature.

According to the results of predicted occurrences it was possible to define conditions suitable for brown trout. The range and the mean of the variables SLO, A_TEM and R_TEM had similar ranges in all extents (Table 5). The suitable range of SLO was between 0.3‰ and 28‰. The suitable conditions of climate variables were found between 5.5 and 17 °C for A_TEM and between 7.3 and 15.7 °C for R_TEM (Table 5).

**Table 5:** Mean and range (minimum–maximum) of environmental variables recognized by models as suitable condition for the presence of brown trout in Iran in different extents in comparison with the original database.

		SLO (‰) (Mo)	SLO (‰) (OD)	A_TEM ( °C) (Mo)	A_TEM ( °C) (OD)	R_TEM ( °C) (Mo)	R_TEM ( °C) (OD)
Small extent	Mean	4	1.7	12.6	17.2	12.4	12.7
	Range	0.3–28	0.0–28.0	5.5–16.6	5.5–27.5	7.3–15.6	6.9–16.5

Medium extent	Mean	4.4	1.7	12.7	17.6	12.5	12.8
	Range	0.3–28	0.0–28.0	5.5–16.8	5.5–27.5	7.3–15.7	6.9–16.5

Large extent	Mean	4.3	1.7	12.9	17.7	12.5	12.9
	Range	0.3–28	0.0–28.0	5.5–17	5.5–27.5	7.5–15.7	6.9–16.6

*Abbreviations*: SLO, stream slope; A_TEM, mean air temperature; R_TEM, range of air temperature; Mo, model; OD, original database.

Finally, the independent validation underlined a good model performance. Out of 15 sites with species absence and 15 sites with species presence, all were predicted correctly by the final model in all three extents.

## Discussion

### Brown trout response to environmental variables

For stream fish, temperature appears to be one of the main determinant factors of spatial distribution (e.g. Buisson et al., 2008, Logez et al., 2012). Freshwater fish as ectothermic animals are particularly sensitive to temperature with effects on their metabolism, breeding, development and growth (Mann, 1996). Accordingly, mean air temperature has been widely shown as an important variable determining fish distributions (e.g. Pont et al., 2005, Buisson et al., 2008, Abdoli and Naderi, 2009), which is in line with the results of this study. The results showed that the brown trout was clearly linked to areas with cold temperatures, indicating a cold-stenotherm behaviour highlighted by many authors such as: Elliott (1994), Pont et al. (2005) and Abdoli and Naderi (2009). Logez et al. (2012) reported eurythermal behavior of brown trout in their study. In contrast to previous studies (Pont et al., 2005, Buisson et al., 2008, Filipe et al., 2013) the importance of the thermal range (range of air temperature) is highlighted in our study. Probably, the range of air temperature was constrained according to restricted variability in other study areas. Logez et al. (2012) highlighted mean air temperature as a dominant parameter determining brown trout distribution but assigned a minor role to thermal range.

Furthermore, slope was of great importance in all extents which is in accordance with Mann (1996), Pont et al. (2005) and Filipe et al. (2013). At the reach scale, river slope is a surrogate for the hydraulics. High slope values are typical for suitable brown trout habitats. Logez et al. (2012) used slope in association with stream size and runoff as a surrogate of stream power which reflects the ability of a stream to move bed substrate and varies with both stream slope and discharge. Consistently, the presence of brown trout increased with increasing stream power in their study. In line with Pont et al. (2005), stream width (bankfull and wetted width) did not show considerable importance for brown trout distribution.

### Brown trout prediction

Literature records the distribution of brown trout in three basins in Iran (Abdoli, 2000, Esmaeili et al., 2010). The results of our modelling framework highlighted these basins as the major area of distribution as most occurrences were predicted there. However, all models also predicted suitable habitats for brown trout outside these areas. In contrast to the described distribution (e.g. Abdoli, 2000, Abdoli and Naderi, 2009) the models predicted brown trout presences in the eastern part of the Caspian Basin as well as in the Tigris Basin (Fig. 3). Some fisheries scientists hypothesised that brown trout may occur in the eastern part of the Caspian Basin which is supported by the results of the models (Fig. 3) but proof is missing so far. Moreover, the available sampling information goes back 20 years when the rainbow trout (*Oncorhynchus mykiss*), an exotic species, was already stocked (Kiabi et al., 1999b) which may additionally impede the proof of former brown trout presence.

The models also identified areas in the Tigris Basin as potential habitats for brown trout. This seems reasonable as brown trout occurs in the upstream parts of Tigris in Turkey (Turan et al., 2011). Additionally, from a biogeographical point of view, the Tigris Basin was the migration route of brown trout to the Namak Basin in palaeo-historic times before the mountains between the basins lifted up (Boulenger, 1896, Berg, 1948–1949, Berg, 1949). Sufficient sampling data for these regions is lacking, especially in upstream regions. This is important to mention, because almost every year new species are being discovered in remote and mountainous regions of Iran (e.g. Coad, 2009, Teimori et al., 2012).

### Brown trout and human impacts

Human activities over recent decades had huge impacts on brown trout occurrences in Iran. Brown trout is currently considered as a vulnerable taxon in Iran (Kiabi et al., 1999a, Mostafavi, 2007). Coad (2000) identified this species as one of the top four threatened freshwater fish species in Iran. Furthermore, Nezami et al. (2000) considered this taxon as endangered. As Akhani et al. (2010) indicated half of the forest in the Caspian Basin was eradicated in recent decades, i.e. from 3.6 million hectares to 1.8 million. In contrast, the extent of agriculture and developed areas has increased over recent decades (Akhani et al., 2010). Beside land cover, the construction of dams represents another constraint in fish species occurrence. The number of dams in Iran has increased dramatically. Currently, there are 607 dams of which 595 were built between 1974 and 2012. Moreover, 559 dams are planned and 142 dams are under construction (http://daminfo.wrm.ir/dam-stats-fa.html). Additionally, water pollution and gravel mining have impacts on water quality, consequently affecting sensitive species (Coad, 1980, Kiabi et al., 1999a, Kiabi et al., 1999b, Abdoli, 2000, Esmaeili et al., 2007, Mostafavi, 2007). A practical example is given for the LiqvanChay River population in the Urmia Basin where trout is now confined to a single river. However, the majority of adequate habitats were destroyed through agriculture and domestication of sheep and goats (Anonymous, 1977). In the Lar River, situated in the Caspian Basin and Karaj River in the Namak Basin, native populations suffered from overfishing by using nets, chemicals and explosives (Surber, 1969). Hence, the native populations of brown trout have declined dramatically. Therefore, our results have important implications for conservation activities and management. The modelling framework has the ability to highlight areas of trout potential occurrence and to identify sites where trout is absent due to habitat degradation. Consequently, based on more detailed future studies effective conservation and restoration measures can be undertaken to maintain and (re)establish brown trout populations.

## Conclusions

The presented modelling framework has proven its suitability to identify brown trout habitats on the Iranian scale. The developed model enables to improve management planning as well as conservation actions. Finally, our model shows, beside a user-friendly applicability, a good performance and prediction accuracy which offers opportunities for further use, e.g. integration into multimetric IBI.
